# Production, Biochemical Characterization, and Kinetic/Thermodynamic Study of Inulinase from *Aspergillus terreus* URM4658

**DOI:** 10.3390/molecules27196418

**Published:** 2022-09-28

**Authors:** Rodrigo Lira de Oliveira, Suzana Pedroza da Silva, Attilio Converti, Tatiana Souza Porto

**Affiliations:** 1 School of Food Engineering, Federal University of Agreste of Pernambuco/UFAPE, Av. Bom Pastor, Boa Vista, s/n, Garanhuns 55296-901, Brazil; 2 Department of Civil, Chemical and Environmental Engineering, Pole of Chemical Engineering, Genoa University, Via Opera Pia 15, 16145 Genoa, Italy; 3 Department of Morphology and Animal Physiology, Federal University of Pernambuco/UFRPE, Av. Dom Manuel de Medeiros, s/n, Recife 52171-900, Brazil

**Keywords:** agro-industrial substrates, *Aspergillus*, inulinase, kinetics, thermodynamics

## Abstract

Inulinases are enzymes involved in the hydrolysis of inulin, which can be used in the food industry to produce high-fructose syrups and fructo-oligosaccharides. For this purpose, different *Aspergillus* strains and substrates were tested for inulinase production by solid-state fermentation, among which *Aspergillus terreus* URM4658 grown on wheat bran showed the highest activity (15.08 U mL^−1^). The inulinase produced by this strain exhibited optimum activity at 60 °C and pH 4.0. A detailed kinetic/thermodynamic study was performed on the inulin hydrolysis reaction and enzyme thermal inactivation. Inulinase was shown to have a high affinity for substrate evidenced by very-low Michaelis constant values (0.78–2.02 mM), which together with a low activation energy (19.59 kJ mol^−1^), indicates good enzyme catalytic potential. Moreover, its long half-life (*t*_1/2_ = 519.86 min) and very high *D*-value (1726.94 min) at 60 °C suggested great thermostability, which was confirmed by the thermodynamic parameters of its thermal denaturation, namely the activation energy of thermal denaturation (E*_d_ = 182.18 kJ mol^−1^) and Gibbs free energy (106.18 ≤ ΔG*_d_ ≤ 111.56 kJ mol^−1^). These results indicate that *A. terreus* URM4658 inulinase is a promising and efficient biocatalyst, which could be fruitfully exploited in long-term industrial applications.

## 1. Introduction

Inulin is a fructan-type oligosaccharide consisting of linear chains of fructose residues linked by *β*(2→1) glycosidic bonds and terminated by a sucrose residue, which is present in considerable amounts in bulbs, tubers, and tuberous roots of many plants such as Jerusalem artichoke, chicory, asparagus, and dahlia [1]. Inulin hydrolysis is performed by inulinases, enzymes that hydrolyze the *β*(2→1) bond releasing fructose, inulo-oligosaccharides, fructo-oligosaccharides (FOS), and glucose [2]. These enzymes are classified, according to their regioselective reaction and mode of action on fructans, as exoinulinases (EC 3.2.1.80) and endoinulinases (EC 3.2.1.7). The enzymes of the former class, hydrolyzing the non-reducing terminal residue of inulin, release fructose and are therefore used in the industry to obtain high-fructose syrup (HFS). On the other hand, endoinulinases break off inulin arbitrarily releasing FOS of different chain length [3,4]. Since FOS have great industrial importance owing to their functional properties and health benefits, recent developments in industrial biotechnology have enabled their large-scale production [5]. In addition, inulinases can be used in bioethanol production from non-conventional feedstocks such as inulin and inulin-containing plant materials [6].

Although inulinases can be obtained from animal, vegetable, and microbial sources, only the microbial ones are commercially exploited since the other sources have low enzyme contents. Moreover, the use of microorganisms to produce inulinases has several advantages, including easy handling, cultivation, and genetic manipulation, as well as high productivity [3]. Several fungi, yeasts, and bacteria produce inulinases in considerable amounts in both submerged fermentation and solid-state fermentation (SSF) [7]. SSF is defined as a process in which microorganisms grow in an environment without free water, or with a very low content of free water. This process is characterized by mimicking the natural habitat of most microorganisms, mainly fungi, using abundantly available agro-industrial residues [2,8]. The main inulinase producers by SSF are the filamentous fungi and yeasts belonging to the genera *Aspergillus* and *Kluyveromyces*, respectively [2].

The biochemical characterization of enzymes is paramount to evaluate their biotechnological potential. Furthermore, the knowledge of their profiles of activity and stability at different pH and temperature values along with their kinetic and thermodynamic parameters can be used to succeed in industrial applications [9]. The kinetic and thermodynamic parameters are very important information for enzymes, whose combined use allows, through correct mathematical tools, predicting their behavior under conditions not yet tested experimentally [10]. The thermodynamic data of enzyme-catalyzed reactions are useful to predict their extent, while enzyme thermal stability, assessed by residual activity, concerns both thermodynamic and kinetic aspects [11]. The activity and thermostability of enzymes are important issues that must be taken into account to assess the economic feasibility of enzyme-based industrial processes, e.g., HFS or FOS production in the specific case of inulinases, thus reinforcing the importance of evaluating the kinetic and thermodynamic parameters [12].

Based on this background, the aims of the present study were the production of *Aspergillus terreus* URM4658 inulinase by SSF using agro-industrial substrates, its biochemical characterization, and the kinetic/thermodynamic study of inulin hydrolysis and inulinase thermal denaturation.

## 2. Results

### 2.1. Inulinase Production and Optimization

Seven *Aspergillus* strains were tested for their ability to produce inulinase by submerged fermentation using inulin from chicory as the substrate. Inulinase activity ranged from 0.63 to 2.20 U mL^−1^ (Table 1), with the highest values being observed for *A. niger* URM5741 (2.20 ± 0.12 U mL^−1^) and *A. terreus* URM4658 (1.27 ± 0.23 U mL^−1^).

These two strains were then studied for enzyme production by solid-state fermentation (SSF) using different agro-industrial wastes (oat bran, soybean meal, and wheat bran) as substrates, whose results are given in Table 2. The maximum activity observed for *A. terreus* URM4658 grown on wheat bran (13.34 ± 0.41 U mL^−1^) confirms its good ability to produce inulinase by SSF on other low-cost substrates already reported for this species [13]. As for the safety of this production, a previous study by our research group demonstrated the *A. terreus* URM4658 strain’s lack of ability to produce aflatoxin in a coconut milk agar medium according to the ammonium vapor test [14].

Inulinases have both inulinase (*I*) and invertase (*S*) activities, whose ratio (*I*/*S*) is a useful tool to identify their predominant action, i.e., values greater than 10^−2^ indicate high inulinase activity, whereas values lower than 10^−4^ high invertase activity [2]. In this regard, the crude extract obtained by SSF on wheat bran showed an *I*/*S* ratio of 0.48, confirming the inulolytic nature of the *A. terreus* URM4658 enzyme.

After the selection of the most suitable fungal strain (*A. terreus* URM4658) and substrate (wheat bran), the influence of the substrate amount, inulin concentration, and moisture content on inulinase production was evaluated according to a 2^3^-full factorial design, whose experimental conditions and results are listed in Table 3.

**Figure 1:**
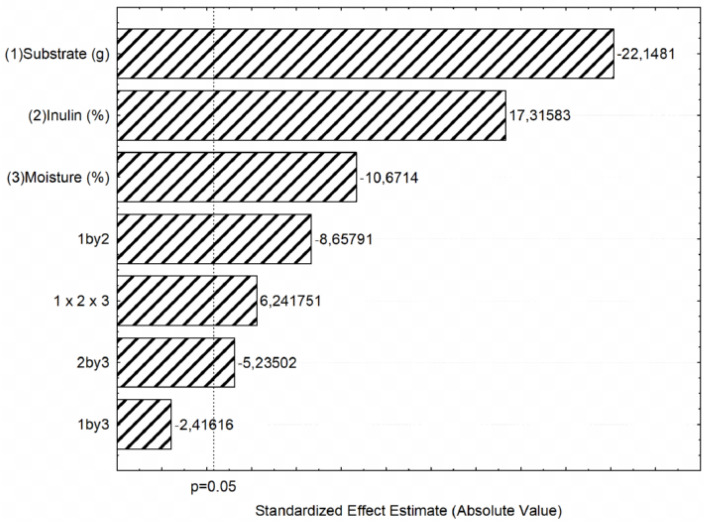
Pareto chart of the standardized effects of substrate amount, inulin concentration, and moisture content on inulinase production by *Aspergillus terreus* URM4658 in solid-state fermentation using wheat bran as a substrate.

The highest inulinase production (14.35 U mL^−1^) was observed in run 3 carried out using 3 g of wheat bran, 7.5% inulin and 50% moisture content at 30 °C. The statistical effects of the variables and interactions were calculated and presented in the Pareto chart shown in Figure 1. All independent variables were statistically significant, however, while the substrate amount and moisture content exerted negative effects on inulinase activity, that of inulin concentration was positive.

For optimization purposes, additional runs were performed according to a central composite rotational design (CCRD), where only the inulin concentration and moisture content were varied, while the substrate (wheat bran) amount was kept constant (3.0 g) (Table 4), since lower substrate masses would hamper the fermentation process.

The highest inulinase activity (15.08 U mL^−1^) was obtained in run 4 carried out using 9.0% inulin concentration and 55% moisture content at 30 °C. Application of the analysis of variance (ANOVA) to the second-order regression model fitted to experimental data (Table 5) indicates that all variables and interactions were statistically significant, except for the linear term of inulin concentration. Although the statistical model exhibited a sufficient prediction ability (R^2^ = 0.878), the ANOVA indicated a significant lack of fit.

### 2.2. Biochemical Characterization

**Figure 2:**
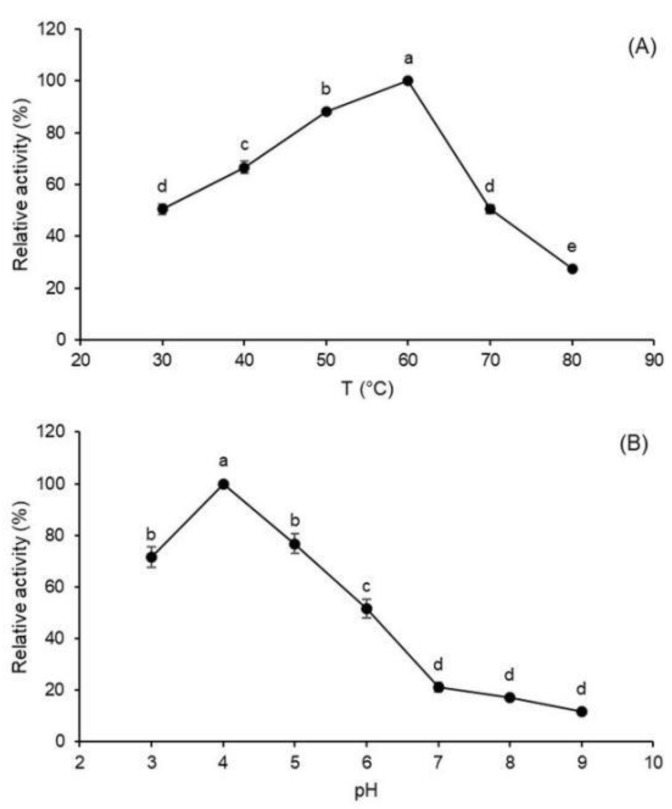
Effect of temperature (**A**) and pH (**B**) on the inulinase activity of *Aspergillus terreus* URM4658 inulinase produced by solid-state fermentation using wheat bran as a substrate. Different letters (a–e) indicate statistically significant differences (*p* < 0.05).

As is known, the rate of any reaction increases by raising the temperature; however, at the same time, progressive enzyme denaturation occurs, which causes activity loss, especially beyond the optimum temperature [15]. The relative inulinase activity of the *A. terreus* crude extract reached a maximum value at 60 °C (Figure 2A) and pH 4.0 (Figure 2B).

Since metal ions can act as activators or inhibitors in enzyme-catalyzed reactions, inulinase activity assays were also carried out on crude extracts from SSF in the presence of different metal ions, whose results are gathered in Table 6. The activity was not stimulated by any of the tested ions, but it was inhibited by Cu^2+^ (35.41%) and almost completely lost using Hg^2+^ (85.21%).

### 2.3. Kinetic and Thermodynamic Parameters of Inulin Hydrolysis

**Figure 3:**
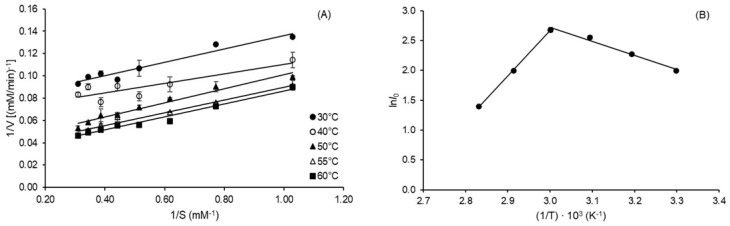
(**A**) Lineweaver–Burk plots for the determination of kinetic parameters of inulin hydrolysis reaction. (**B**) Arrhenius type-plot used to estimate the activation energy (E*_a_) of inulin hydrolysis catalyzed by inulinase from *Aspergillus terreus* URM4658 and the standard enthalpy variation of the enzyme unfolding equilibrium (∆*H*°_u_). *I_0_* is the starting inulinase activity and *T* is the absolute temperature.

The temperature-dependent kinetic parameters of inulin hydrolysis by *A. terreus* inulinase, namely the Michaelis constant (*K_m_*), the maximum reaction rate (*V_max_*), and the catalytic constant (*k_cat_*), were estimated with a good correlation (0.929 ≤ R^2^ ≤ 0.974) from Lineweaver–Burk plots (Figure 3A) and listed in Table 7.

Increases in *K_m_* from 0.78 to 2.02 mM, *V_max_* from 13.09 to 35.09 mM min^−1^ and *k_cat_* from 2.49 to 6.68 min^−1^ were observed with increasing temperature from 30 to 60 °C. The values of the activation energy of inulin hydrolysis (E*_a_ = 19.54 ± 1.10 kJ mol^−1^) and the standard enthalpy variation of the enzyme unfolding equilibrium (∆*H*°_u_ = 82.49 ± 1.10 kJ mol^−1^) were estimated from the slopes of straight lines of the Arrhenius type-plot shown in Figure 3B with excellent correlation (0.982 ≤ R^2^ ≤ 0.996). The other thermodynamic parameters of inulin hydrolysis, namely activation enthalpy (ΔH*), Gibbs free energy (ΔG*), and entropy (ΔS*), calculated as described in Section 4.6.1, ranged from 16.82 to 17.07 kJ mol^−1^, 82.31 to 87.98 kJ mol^−1^, and −208.32 to −213.60 J K^−1^ mol^−1^, respectively. The temperature quotient (*Q_10_*), which indicates the activity increase resulting from a 10 °C rise in temperature and is often used to identify the factor controlling a reaction, was determined for a more accurate understanding of the influence of temperature on inulin hydrolysis. It can be seen in Table 7 that this parameter remained almost constant (1.07–1.08) in the investigated range of temperature (30–60 °C).

### 2.4. Kinetic and Thermodynamic Parameters of Inulinase Thermal Denaturation

**Figure 4:**
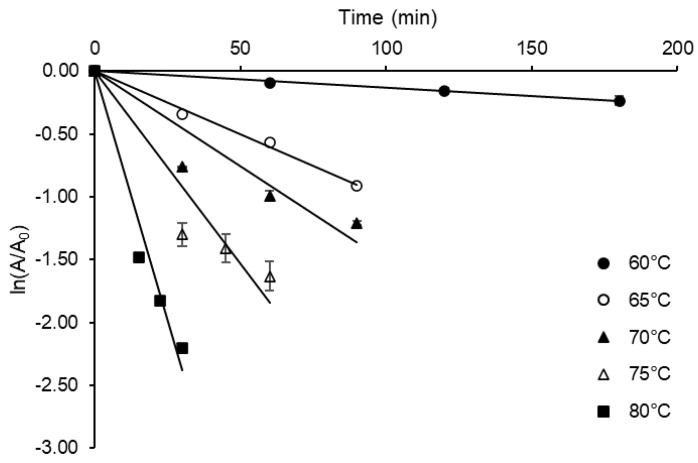
Semi-log plots of the residual activity coefficient (*I*/*I_0_*) of *Aspergillus terreus* URM4658 inulinase versus time.

In addition to a better understanding of enzyme-catalyzed reactions, a kinetic and thermodynamic study can also provide insights into the enzyme denaturation process, which represents one of the main restrictions in many industrial applications of enzyme processes [16]. The thermal denaturation of *A. terreus* inulinase was investigated through residual activity tests carried out in the range of 60–80 °C, which allowed to estimate, with good correlation (0.959 ≤ R^2^ ≤ 0.997), the first-order denaturation rate constant (*k_d_*) at each temperature from the semi-log plot of the activity coefficient [ln(*I*/*I_0_*)] versus time (Figure 4).

The *k_d_* values listed in Table 8 are consistent with the typical enzyme denaturation behavior characterized by a progressive increase in this parameter with temperature (from 0.0013 to 0.0794 min^−1^). The other kinetic parameters calculated from the *k_d_* value at 60 °C (*t*_1/2_ = 519.86 min; *D*-value = 1726.94 min) are a good indicator of the high enzyme thermostability. The thermal resistance constant (*Z*-value), estimated with good correlation (R^2^ = 0.933) from the slope of the graph of log*D* versus temperature, was shown to be 12.39 °C.

**Figure 5:**
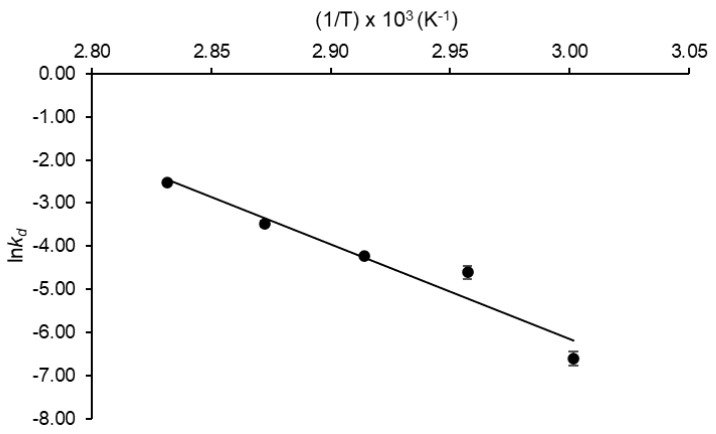
Arrhenius-type plot used to estimate the activation energy of *Aspergillus terreus* URM4658 inulinase thermal denaturation.

An activation energy of enzyme thermal denaturation (E*_d_) as high as 182.18 kJ mol^−1^ was estimated with satisfactory correlation (R^2^ = 0.938) from the slope of the Arrhenius-type plot illustrated in Figure 5. As expected from Equation (1) presented in Section 4.6.1, high values were also calculated for the activation enthalpy of enzyme denaturation (179.24 ≤ ΔH*_d_ ≤ 179.40 kJ mol^−1^). As for the other thermodynamic parameters, the activation entropy (ΔS*_d_) and Gibbs free energy (ΔG*_d_) of this event varied in the ranges 203.64–212.08 J mol^−1^ K^−1^ and 106.44–111.56 kJ mol^−1^, respectively.

## 3. Discussion

The results of *A. terreus* URM4658 inulinase production by solid-state fermentations (SSFs) performed according to a 2^3^-full factorial design showed a statistically significant positive effect of inulin concentration on enzyme activity. This effect, already observed for inulinase production by *Kluyveromyces* sp. in SSF [17], can be ascribed to the fact that inulin is not only a carbon source but also acts as an inducer of inulinase expression; therefore, for the same reason, excessively high inulin concentrations can lead to catabolic repression [3,17]. After optimization was performed according to a central composite rotational design (CCRD), the corresponding inulin hydrolysis activity per unit dry mass of substrate (177.83 U gds^−1^) was more than twice that reported by Singh et al. [18] for *Penicillium oxalicum* inulinase production using corn bran as a substrate (77.95 U gds^−1^).

As for the characteristics of the enzyme, its optimum temperature (60 °C) is the same as that reported for *Aspergillus fumigatus* [19], *Aspergillus niger* [20], and *Aspergillus tubingensis* [21] inulinases. With an optimum pH of 4.0, the enzyme proved to be resistant to acidic conditions, a feature of great interest for high-fructose syrup (HFS) production, because it reduces the risk of microbial contamination especially during continuous operation [22]. The inhibition of inulinase activity by Cu^2+^ can be explained by the possible interaction of this ion with SH-groups of membrane proteins and internal environmental changes in tryptophan residues, which are responsible for tertiary structure variations [23,24]. On the other hand, the almost complete suppression of activity by Hg^2+^ may have been due to its ability to react with thiol groups converting them into mercaptides, as well as with histidine and tryptophan residues [25]. A similar strong inhibition by Hg^2+^ was observed for pre-purified or purified inulinases from *A. niger* [26], *Penicillium* sp. [27], and *Ulocladium atrum* [28], for which residual activities of only 9.0, 21.59, and 18.6% were reported, respectively.

The kinetic results of hydrolytic activity indicate that the enzyme affinity for substrate, expressed by the value of the Michaelis constant (*K_m_*), reached a maximum at 30 °C, while, as expected, the maximum reaction rate (*V_max_*) and catalytic constant or turnover number (*k_cat_*) did so at 60 °C. In general, the *A. terreus* URM4658 inulinase showed a good affinity for substrates at all tested temperatures, which was higher than that reported for purified *A. terreus* exoinulinase (*K_m_* = 11 mM) [29].

The very low value of the activation energy of inulin hydrolysis (19.59 ± 1.10 kJ mol^−1^) indicates that low energy is required to form the activated complex, which is an indicator of the high catalytic potential of this inulinase. It was lower than those of inulinases from other *Aspergillus* strains, such as *A. niger* (25.20–60.95 kJ mol^−1^) [30], *A. welwitschiae* MN056175 (21.82 kJ mol^−1^) [31], *A. awamori* (32.56 kJ mol^−1^) [32], and a marine strain of *A. terreus* (28.41 kJ mol^−1^) [33]. On the other hand, the relatively high value of standard enthalpy variation of enzyme unfolding equilibrium (*∆H*°_u_) (82.49 ± 1.10 kJ mol^−1^) highlights an unfavorable biocatalyst unfolding and then good performance of the process [16]. To the best of our knowledge, this parameter has not been studied for inulin hydrolysis reaction to date; therefore, a comparison with other studies is not possible.

The very low values of the activation enthalpy of enzyme-catalyzed reaction (ΔH*) calculated at all tested temperatures (16.82–17.07 kJ mol^−1^) indicate that the formation of the transition state or activated enzyme–substrate complex occurred effectively. Moreover, the slight decrease in ΔH* resulting from a temperature rise means that this formation was more effective at higher temperatures. As is known, the activation entropy of an enzyme-catalyzed reaction (ΔS*) is related to the order (rigidity) degree of the activated enzyme–substrate complex [10]. The negative and large ΔS* values observed in the tested temperature range suggest that the transition state had a more ordered structure than the reacting system, i.e., its formation implied a reduction in freedom degree [34]. The Gibbs free energy of activation (ΔG*) represents the free energy difference from the reactants to the transition state, which is the minimum energy to activate the reaction and basically tells how fast the reaction could be. Moreover, it is the most suitable parameter to evaluate the feasibility of a chemical reaction, in that, the lower its value is, the more spontaneous the conversion of the enzyme–substrate complex into products is [35]. Therefore, the slight increase in ΔG* observed with the increasing temperature from 30 to 60 °C highlights a temperature-dependent decrease in the spontaneity of inulin hydrolysis. Similar qualitative behavior was reported by Silva et al. [10] for pectin hydrolysis catalyzed by free and immobilized pectinases from *Aspergillus aculeatus*.

Commonly, enzyme-catalyzed reactions are characterized by temperature quotient (*Q_10_*) values between 1 and 2 indicating that the reaction is controlled by temperature, while deviations from this range indicate the predominance of other factors [36]. The *Q_10_* values calculated in the present study were approximately 1.07 and 1.08 in the temperature range 30–60 °C, which means that the inulin hydrolysis was kinetically controlled by temperature. Qualitatively similar temperature-dependence was observed for inulinases from *A. niger* (1.31 ≤ *Q_10_* ≤ 1.72) at 30–60 °C [20] and *Penicillium oxalicum* (1.22 ≤ *Q_10_* ≤ 1.24) at 50–60 °C [37].

The values of kinetic parameters directly affected by inulinase thermal denaturation listed in Table 8, i.e., the half-life (*t*_1/2_) and decimal reduction time (*D*-value), revealed that *A. terreus* inulinase was more stable than the inulinases from *A. welwitschiae* (*t*_1/2_ = 133.22 min; *D*-value = 509.08 min) [31], *Aspergillus tritici* (*t*_1/2_ = 129.0 min; *D*-value = 428.0 min) [38], and *A. niger* (*t*_1/2_ = 77.88 min; *D*-value = 256.41 min) [39] at 60 °C. The thermal resistance constant (*Z*-value) (12.39 °C) was very close to that of purified *A. tritici* endoinulinase (11.52 °C) [38] and lower than that of free (16.20 °C) and immobilized (21.55 °C) inulinase from *P. oxalicum* [37]. In general, low *Z*-values such as that obtained in this study indicate that the enzyme is more sensitive to a temperature rise than to the duration of thermal treatment [40].

As for the thermodynamic parameters of enzyme thermal denaturation, the activation enthalpy of this phenomenon (ΔH*_d_) corresponds to the total amount of energy required to denature an enzyme through the disruption of non-covalent bonds; therefore, higher values of ΔH*_d_ and activation energy (E*_d_) indicate stronger intramolecular stabilizing forces and less extended conformation [34]. The E*_d_ value obtained in the present study is much higher than those reported for other *Aspergillus* inulinases, e.g., those from *A. welwitschiae* (73.21 kJ·mol^−1^) [31] and *A. niger* (26.45 kJ·mol^−1^) [39], which points out a better thermostability of the enzyme under investigation, considering that a higher energetic barrier must be overcome to denaturate it. It has been estimated that the energy required to remove a -CH_2_ moiety from a hydrophobic bond is approximately 5.4 kJ mol^−1^ [41]; therefore, the range of ΔH*_d_ values listed in Table 8 (195.78–195.95 kJ mol^−1^) suggest that no less than 36 non-covalent bonds were disrupted during *A. terreus* inulinase denaturation.

Since the denaturation process is characterized by the opening of the enzyme structure, it is usually accompanied by an increase in the disorder and randomness degree evidenced by positive activation entropy (ΔS*_d_) values such as those estimated in this study. On the other hand, the activation free energy (ΔG*_d_), which combines both enthalpy and entropy contributions, is especially useful to obtain information on the spontaneity of thermal denaturation. High and positive values such as those observed in this work indicate special resistance to the denaturation process, confirming the thermostability indicated by the other kinetic and thermodynamic parameters. These values (106.44–111.56 kJ mol^−1^) are closer to those reported for *P. oxalicum* exoinulinase (104.14 ≤ ΔG*_d_ ≤ 113.82 kJ mol^−1^) [42] and not so far from those of other industrial carbohydrate-hydrolyzing enzymes, including *Aspergillus tamarii* β-fructofuranosidase (97.95 ≤ ΔG*_d_ ≤ 108.78 kJ mol^−1^) [34] and *Aspergillus awamori* amylase (85.96 ≤ ΔG*_d_ ≤ 95.74 kJ mol^−1^) [43].

## 4. Materials and Methods

### 4.1. Microorganisms and Inoculum Preparation

Seven fungal strains belonging to the genus *Aspergillus*, provided by the “Micoteca-URM” of the Mycology Department, Centre of Biosciences, of the Federal University of Pernambuco (UFPE), Recife, PE, Brazil, were tested as inulinase producers. The strains used were *A. aculeatus* URM4953, *A. japonicus* URM5620, *A. phoenicis* URM4924, *A. niger* URM5741, *A. niveus* URM5870, *A. tamarii* URM4634, and *A. terreus* URM4658. All strains were maintained in Czapek Dox Agar medium and mineral oil at room temperature (25 ± 0.5 °C) and grown for 3 days in reactivation broth composed of bacteriological peptone (1.0%, *w v*^−1^), meat extract (0.3%, *w v*^−1^), and glucose (2.0%, *w v*^−1^). The microorganisms were then inoculated onto Czapek Dox Agar medium for 7 days at 30 °C for sporulation. The spores were finally collected using a sterilized solution containing NaCl (0.9%, *w v*^−1^) and Tween 80 (0.01%).

### 4.2. Screening of Inulinase Production by Submerged Fermentation

The strain screening was performed by submerged fermentation using food-grade inulin from chicory as a substrate (Frutafit ^®^ CLR, Sensus, Roosendaal, The Netherlands). Fermentations were performed in 250 mL Erlenmeyer flasks containing 50 mL of culture medium, whose composition, adapted according to Skowronek and Fiedurek [44], was as follows (%, *w v*^−1^): inulin, 1.0; yeast extract, 0.3; NaNO_3_, 0.2; K_2_HPO_4_; 0.1; KCl, 0.05; MgCl_2_.6H_2_O, 0.05; FeSO_4_, 0.001; and CuSO_4_, 0.0005. The initial pH was adjusted to 6.0, and an inoculum concentration of 10^5^ spores mL^−1^ was used. Submerged fermentations were performed at 30 °C under the agitation of 150 rpm for 72 h. At the end of fermentations, the cultures were vacuum filtered through a qualitative filter paper for cell pellet removal, and the filtrate was analyzed and stored at −22 °C.

### 4.3. Production of Inulinase by Solid-State Fermentation

The selected strains were used for inulinase production by solid-state fermentation (SSF) using different agro-industrial substrates, namely wheat bran, oat bran, and soybean meal. SSF was performed at 30 °C in 125 mL Erlenmeyer flasks containing 5.0 g of substrate for 72 h. The moisture content of substrate was adjusted to 60% using a nutrition solution (5.0% inulin and 0.5% yeast extract) and spore suspension (10^7^ spores g^−1^). After addition of 7.5 mL of acetate buffer (0.1 M; pH 5.0) per g of fermented material, the inulinase-containing suspension was homogenized in an orbital shaker for 90 min at 120 rpm. Solids were removed by centrifugation at 5000 rpm for 15 min at 4 °C, and the supernatant was analyzed and stored at −22 °C.

After the selection of the best strain (*A. terreus* URM4658) and substrate (wheat bran) for inulinase production by SSF, the influence of the main variables involved in the process was evaluated through a 2^3^-full factorial design, in which the substrate amount (3.0, 5.0, and 7.0 g), inulin concentration (2.5, 5.0, and 7.5%), and moisture content (50, 60, and 70%) were selected as the independent variables. For optimization purposes, additional runs were then carried out according to a central composite rotational design (CCRD) at a constant amount of substrate (3.0 g), but varying the levels of the other two independent variables. All statistical analyses were performed using the software package Statistica 7.0 (Statsoft Inc., Tulsa, OK, USA). The adjustment of a quadratic model to the experimental results obtained from the runs performed according to the CCRD was checked by the Fischer’s test. The fitting ability of the model was assessed by the coefficient of determination (R^2^) and the analysis of variance (ANOVA).

### 4.4. Analytical Determinations

#### 4.4.1. Enzyme Assays

To determine the inulinase activity (*I*) 0.1 mL of the enzyme extract and 0.9 mL of inulin (1.0% in 0.1 M acetate buffer, pH 5.0) were incubated at 55 °C for 10 min. The reducing sugars in the hydrolysate were quantified by the Miller method [45] using fructose as a standard. One unit of inulinase was defined as the amount of enzyme that produced one micromole of fructose per minute, under standard assay conditions. For determination of invertase activity (*S*) and calculation of the *I*/*S* ratio, the reaction was performed under the same experimental conditions, but replacing inulin with sucrose.

#### 4.4.2. Protein Determination

The total protein concentration was determined by the Bradford method [46] using bovine serum albumin (BSA) as the standard.

### 4.5. Biochemical Characterization

#### 4.5.1. Effect of Temperature and pH on Inulinase Activity

The effect of temperature on inulinase activity was investigated by hydrolytic activity tests carried out at different temperatures (30–80 °C) and constant pH (5.0). On other hand, the influence of pH was evaluated by activity tests using inulin as the substrate, diluted in different buffers (0.1 M), namely citrate (3.0 ≤ pH ≤ 4.0), acetate (4.0 ≤ pH ≤ 5.0), citrate–phosphate (5.0 ≤ pH ≤ 7.0), and Tris-HCl (7.0 ≤ pH ≤ 9.0) buffers, at constant temperature (55 °C).

#### 4.5.2. Effect of Metal Ions on Inulinase Activity

The effect of metal ions as inhibitors or activators of inulinase activity was studied by incubating equal volumes of each ion solution and enzyme extract at 25 ± 0.5 °C for 30 min, and the residual hydrolytic activity was measured. For this purpose, the following salts were used at a concentration of 10 mM in 0.1 M sodium acetate buffer (pH 5.0): zinc sulphate heptahydrate (ZnSO_4_.7H_2_O), magnesium sulphate heptahydrate (MgSO_4_.7H_2_O), copper (II) sulphate pentahydrate (CuSO_4_.5H_2_O), iron (II) sulphate heptahydrate (FeSO_4_.7H_2_O), calcium chloride (CaCl_2_), mercury (II) chloride tetrahydrate (HgCl_2_.4H_2_O), potassium chloride (KCl), and sodium chloride (NaCl).

### 4.6. Kinetic/Thermodynamic Study

#### 4.6.1. Kinetic and Thermodynamic Parameters of Inulin Hydrolysis

The kinetic parameters of inulin hydrolysis, namely the Michaelis constant (*K_m_*) and maximum reaction rate (*V_max_*), were estimated by Lineweaver–Burk plots. For this purpose, inulinase activity assays were performed using different initial substrate concentrations (6.0 ≤ *S_0_* ≤ 20.0 mg mL^−1^; 0.65 ≤ *S_0_* ≤ 3.24 mM) at a constant pH (5.0) and different temperatures (30, 40, 50, 55, and 60 °C). The catalytic constant (*k_cat_*) at each temperature was calculated as the ratio of *V_max_* to the protein concentration determined according to the Bradford method. The activation energy of the enzyme-catalyzed reaction (E*_a_) and enthalpy variation of the unfolding equilibrium (*∆H*^°^_u_) were estimated from the slopes of the right (*a*) and left (*a*’) straight lines of the Arrhenius type-plot of ln*I_0_* versus 1/*T*, respectively, as described in Equations (1) and (2) and reported by Converti et al. [16]:

(1) $$a=\frac{-E_{a}}{R}\;$$

(2) $$a\prime =\frac{\Delta H_{u}^{{}^{\circ}}-E_{a}}{R}\;$$

where *I_0_* is the starting inulinase activity, *R* is the gas constant (8.314 J mol^−1^ K^−1^), and *T* is the absolute temperature.

The other thermodynamic parameters of the enzyme-catalyzed reaction, i.e., the activation enthalpy (∆H*), Gibbs free energy (∆G*), and entropy (∆S*), were calculated using the following equations at the same temperatures used for the determination of the kinetic parameters:

(3) $$\Delta H^{*}=E_{a}^{*}-RT\;$$

(4) $$\Delta G^{*}=-RT\ln\left(\frac{k_{cat}h}{k_{b}T}\right)\;$$

(5) $$\Delta S^{*}=\frac{\Delta H^{*}-\Delta G^{*}}{T}\;$$

where *h* is the Planck constant (6.626 × 10^−34^ J s) and *k_b_* is the Boltzmann constant (1.381 × 10^−23^ J K^−1^).

The temperature quotient (*Q_10_*), which is the factor by which the enzyme activity increases due to a 10 °C temperature increase, was calculated by the following equation proposed by Dixon and Webb [47]:

(6) $$Q_{10}=\mathrm{anti}\;\log\left(\frac{E_{a}^{*}\times 10}{RT^{2}}\right)$$

#### 4.6.2. Kinetic and Thermodynamic Parameters of Inulinase Thermal Denaturation

As is known, an increase in temperature promotes not only the enzyme unfolding but also the subsequent denaturation of its structure. The enzyme thermoinactivation phenomenon can be treated as a first-order reaction, taking into account the progressive loss of enzyme activity over time [16]:

(7) $$\frac{dA}{dt}=-k_{d}\;\cdot \;A\;$$

where *A* is the enzyme activity, *t* is the time of exposure at a given temperature and *k_d_* is the first-order denaturation constant.

In our case, the following linearized form of Equation (7) was used to estimate *k_d_* from the slopes of the straight lines obtained by plotting the experimental data of ln(*I*/*I_0_*) versus time in the temperature range of 60–80 °C:

(8) $$\ln\left(\frac{I}{I_{0}}\right)=-k_{d}\;\cdot \;t\;$$

where *I_0_* is the starting inulinase activity at *t* = 0.

The *k_d_* values determined at each temperature were plotted according to an Arrhenius type-plot (ln*k_d_* versus 1/*T*), and the activation energy of the enzyme thermal denaturation (E*_d_) was estimated from the slope of the resulting straight line. The activation enthalpy (∆H*_d_), Gibbs free energy (∆G*_d_), and entropy (∆S*_d_) of enzyme denaturation were then calculated by Equations (3)–(5), but replacing *k_cat_* with *k_d_* and E*_a_ with E*_d_.

Equations (9) and (10) allowed to determine two additional kinetic parameters of the denaturation process, namely the half-life (*t*_1/2_) and decimal reduction time (*D*-value), which are defined as the times after which the enzyme activity is reduced to one half and one tenth of the initial value, respectively, at a given temperature:

(9) $$\;t_{1/2}=\frac{\ln2}{k_{d}}\;$$

(10) $$D-\mathrm{value}=\frac{\ln10}{k_{d}}\;$$

Finally, using the *D*-values obtained at different temperatures, the thermal resistance constant (*Z*-value) was estimated from the slope of the thermal–death–time plot of log*D* versus *T* (°C). The *Z*-value corresponded to the temperature increase required to reduce the *D*-value by one logarithmic unit, i.e., by 90% [16].

## 5. Conclusions

*A. terreus* URM4658 and wheat bran were selected as the best inulinase producer and substrate for solid-state fermentation devoted to inulinase production. The maximum inulinase activity was observed at 60 °C and pH 4.0. The enzyme showed a good affinity for inulin as a substrate, as evidenced by low *K_m_* values at all tested temperatures (30–60 °C). An in-depth kinetic/thermodynamic study of inulin hydrolysis provided important information and contributed to a better understanding of this reaction, while the kinetic and thermodynamic parameters of thermal denaturation evidenced the high thermostability of this enzyme at 60 °C (*t*_1/2_ = 519.86 min; *D*-value = 1726.94 min; ΔG*_d_ = 111.56 kJ mol^−1^). Overall, *A. terreus* inulinase proved to be a promising efficient biocatalyst for potential long-term use in industrial applications such as the productions of high-fructose syrup and fructo-oligosaccharides. Further investigation involving its regioselectivity, purification, and immobilization should be performed for a complete bioprocess development.

## Figures and Tables

**Table 1:** Inulinase activity of crude extracts obtained by submerged fermentation using different *Aspergillus* strains and inulin from chicory as a substrate.

Strain	Inulinase Activity (U mL^−1^)
*A. aculeatus* URM4953	0.84 ± 0.12 ^b,c^
*A. japonicus* URM5620	0.93 ± 0.12 ^b,c^
*A. niger* URM5741	2.20 ± 0.12 ^a^
*A. niveus* URM5870	0.63 ± 0.18 ^c^
*A. phoenicis* URM4924	0.84 ± 0.11 ^b,c^
*A. tamarii* URM4634	0.68 ± 0.05 ^c^
*A. terreus* URM4658	1.27 ± 0.23 ^b^

The experiments were performed in triplicate, and the results expressed as means ± standard deviations. Different superscript letters (a–c) indicate a statistically significant difference among values (*p* < 0.05).

**Table 2:** Inulinase production by *Aspergillus niger* URM5741 and *Aspergillus terreus* URM4658 after 72 h of solid-state fermentation using different agro-industrial substrates.

**Substrate**	**Inulinase Activity (U mL^−1^)**	
	***A. niger* URM5741**	***A. terreus* URM4658**
Oat bran	5.57 ± 0.36 ^c^	10.26 ± 0.05 ^c^
Soybean meal	10.41 ± 0.87 ^a^	11.83 ± 0.15 ^b^
Wheat bran	8.51 ± 0.92 ^b^	13.34 ± 0.41 ^a^

The experiments were performed in triplicate and the results expressed as means ± standard deviations. Different superscript letters (a–c) in the same column indicate a statistically significant difference among values (*p* < 0.05).

**Table 3:** Experimental conditions and results of inulinase production by *Aspergillus terreus* URM4658 in solid-state fermentation carried out at 30 °C for 72 h using wheat bran as a substrate. Runs were carried out according to a 2^3^-full factorial design.

Run	Substrate Amount (g)	Inulin Concentration (%)	Moisture Content (%)	Inulinase Activity (U mL^−1^)
1	3	2.5	50	10.97
2	7	2.5	50	10.54
3	3	7.5	50	14.35
4	7	7.5	50	11.23
5	3	2.5	70	11.26
6	7	2.5	70	9.27
7	3	7.5	70	12.57
8	7	7.5	70	10.14
9	5	5.0	60	13.62
10	5	5.0	60	13.22
11	5	5.0	60	13.12

**Table 4:** Experimental conditions and results of inulinase production by *Aspergillus terreus* URM4658 in solid-state fermentation using wheat bran as substrate. Runs were performed according to a central composite rotational design.

Run	Inulin Concentration (%)	Moisture Content (%)	Inulinase Activity (U mL^−1^)
1	6.0	45	11.30
2	6.0	55	12.97
3	9.0	45	11.63
4	9.0	55	15.08
5	5.4	50	14.68
6	9.6	50	13.91
7	7.5	43	10.72
8	7.5	57	13.37
9	7.5	50	12.61
10	7.5	50	12.75
11	7.5	50	12.97

**Table 5:** Results of ANOVA apply to the second-order regression model fitted to experimental data of inulinase production by *Aspergillus terreus* URM4658 in solid-state fermentation using wheat bran as a substrate. Runs were performed according to the central composite rotational design outlined in Table 4.

Source	Sum of Squares	Degrees of Freedom	Mean Square	*F*-Value	*p*-Value ^a^
(1) Inulin concentration (L)	0.22	1	0.22	6.86	0.120
Inulin concentration (Q)	2.41	1	2.40	72.06	0.013
(2) Moisture content (L)	9.83	1	9.83	294.33	0.003
Moisture content (Q)	1.26	1	1.26	37.79	0.025
1 (L) × 2 (L)	0.79	1	0.79	23.69	0.040
Lack of fit	2.14	3	0.71	21.35	0.045
Pure error	0.06	2	0.03		
Total SS	18.19	10			

^a^ Statistically significant for *p*-values ≤ 0.05. L = linear term; Q = quadratic term; SS = sum of squares.

**Table 6:** Effect of metal ions at 10 mM concentration on the hydrolytic activity of inulinase from *Aspergillus terreus* URM4658.

Metal Ion	Residual Inulinase Activity (%)
Ca^2+^	98.31 ± 0.10 ^a^
Cu^2+^	64.59 ± 0.27 ^d^
Fe^2+^	92.40 ± 0.45 ^a,b^
Hg^2+^	14.79 ± 1.50 ^e^
K^+^	87.84 ± 2.26 ^b,c^
Mg^2+^	90.34 ± 1.21 ^a,b^
Na^+^	79.91 ± 4.76 ^c^
Zn^2+^	86.59 ± 2.78 ^b,c^

The experiments were performed in triplicate and the results expressed as means ± standard deviations. Different superscript letters (a–e) indicate a statistically significant difference among values (*p* < 0.05).

**Table 7:** Kinetic and thermodynamic parameters of inulin hydrolysis catalyzed by inulinase from *Aspergillus terreus* URM4658 at different temperatures.

Parameter	Temperature (°C)				
	30	40	50	55	60
^a^*K_m_* (mM)	0.78 ± 0.03 ^C^	0.86 ± 0.10 ^C^	1.68 ± 0.08 ^B^	1.72 ± 0.05 ^A^^,^^B^	2.02 ± 0.12 ^A^
^b^*V_max_* (mM min^−1^)	13.09 ± 0.20 ^C^	16.03 ± 0.89 ^C^	26.46 ± 0.29 ^B^	30.30 ± 0.36 ^B^	35.09 ± 1.57 ^A^
^c^*k_cat_* (min^−1^)	2.49 ± 0.04 ^C^	3.05 ± 0.17 ^C^	5.03 ± 0.06 ^B^	5.76 ± 0.07 ^B^	6.68 ± 0.30 ^A^
*R* ^2^	0.939	0.929	0.964	0.971	0.974
^d^ *Q_10_*	1.08 ± 0.004 ^A^	1.08 ± 0.004 ^A^	1.08 ± 0.004 ^A^	1.07 ± 0.004 ^A^	1.07 ± 0.004 ^A^
^e^ ΔH* (kJ mol^−1^)	17.07 ± 1.10 ^A^	16.99 ± 1.10 ^A^	16.90 ± 1.10 ^A^	16.86 ± 1.10 ^A^	16.82 ± 1.10 ^A^
^f^ ΔG* (kJ mol^−1^)	82.31 ± 0.03 ^E^	84.58 ± 0.14 ^D^	86.02 ± 0.03 ^C^	87.02 ± 0.03 ^B^	87.98 ± 0.12 ^A^
^g^ ΔS* (J K^−1^ mol^−1^)	−215.20 ± 3.76 ^A^	−215.85 ± 3.06 ^A^	−213.88 ± 3.31 ^A^	−213.80 ± 3.46 ^A^	−213.60 ± 3.68 ^A^

^a^ Michaelis constant; ^b^ maximum reaction rate; ^c^ turnover number; ^d^ temperature quotient; ^e^ activation enthalpy; ^f^ activation Gibbs free energy; ^g^ activation entropy. Different superscript letters (A–E) indicate statistically significant differences among values (*p* < 0.05).

**Table 8:** Kinetic and thermodynamic parameters of the thermal denaturation of inulinase produced by *Aspergillus terreus* URM4658 in solid-state fermentation using wheat bran as a substrate.

Parameter	*T* (°C)				
	60	65	70	75	80
^a^*k*_d_ (min^−1^)	0.0013 ± 0.0002	0.0100 ± 0.0015	0.0152 ± 0.0007	0.0307 ± 0.0022	0.0794 ± 0.0007
*R* ^2^	0.997	0.997	0.959	0.970	0.988
^b^*t*_1/2_ (min)	519.86 ± 81.69	70.16 ± 10.91	47.21 ± 2.27	22.64 ± 1.67	8.73 ± 0.08
^c^*D*-value (min)	1726.94 ± 271.36	233.08 ± 36.26	161.59 ± 7.54	75.21 ± 5.54	29.00 ± 0.26
^d^*Z*-value (°C)	12.39 ± 0.14				
^e^E*_d_ (kJ mol^−1^)	182.18 ± 2.11				
^f^ ΔG*_d_ (kJ mol^−1^)	111.56 ± 0.44	107.64 ± 0.44	108.25 ± 0.01	107.65 ± 0.21	106.44 ± 0.03
^g^ ΔH*_d_ (kJ mol^−1^)	179.40 ± 2.11	179.36 ± 2.11	179.32 ± 2.11	179.28 ± 2.11	179.24 ± 2.11
^h^ ΔS*_d_ (J mol^−1^ K^−1^)	203.64 ± 5.02	212.08 ± 7.54	207.10 ± 6.11	205.73 ± 5.45	206.11 ± 6.05

^a^ First-order denaturation rate constant; ^b^ half-life; ^c^ decimal reduction time; ^d^ thermal resistance constant; ^e^ activation energy; ^f^ activation Gibbs free energy; ^g^ activation enthalpy; and ^h^ activation entropy.

## Data Availability

Raw data are available upon request.
